# Edaravone Improves the Efficacy of Metformin Against Diabetic Nephropathy by Attenuating Inflammation, Oxidative Stress, and Apoptosis with Associated Modulation of MAPK/NF-κB1 and AKT/NRF2 Pathways in Rats

**DOI:** 10.3390/biology15211988

**Published:** 2026-11-03

**Authors:** Bodour S. Rajab, Ahmed M. El-Sebaey, Anmar A. Khan, Talat A. Albukhari, Bassem Refaat, Hatem A. Sembawa, Rehab M. Bagadood, Eyad T. Albukhari, Nani Nasreldin, Mohamed E. El-Boshy

**Affiliations:** 1 Department of Clinical Laboratory Sciences, Faculty of Applied Medical Sciences, Umm Al-Qura University, Makkah 21955, Saudi Arabia; 2 Department of Clinical Pathology, Faculty of Veterinary Medicine, Mansoura University, Mansoura 35516, Egypt; dr_sebaey@mans.edu.eg; 3 Department of Hematology and Immunology, Faculty of Medicine, Umm Al-Qura University, Makkah 21955, Saudi Arabia; 4 Department of Basic Medical Sciences, Makkah National College, Makkah 24235, Saudi Arabia; b.refaat@mnc.edu.sa; 5 Department of Surgery, Faculty of Medicine, Umm Al-Qura University, Makkah 21955, Saudi Arabia; 6 Faculty of Medicine, Umm Al-Qura University, Makkah 21955, Saudi Arabia; 7 Department of Pathology and Clinical Pathology, Faculty of Veterinary Medicine, New Valley University, El-Kharga 72511, Egypt

**Keywords:** edaravone, metformin, diabetic nephropathy, oxidative stress, TGF-β/NF-κB1, *MAPK-1*/NRF2

## Abstract

The present study investigated the protective effects of edaravone (EDA), metformin (Met), and their combined treatment against diabetic nephropathy (DN) in rats. Sixteen normal non-diabetic rats were allocated to the negative control (NC) and EDA-treated control (EDA10; 10 mg/kg/day) groups and received a standard diet throughout the study. Type 2 diabetes was induced by feeding male Wistar rats (*n* = 40) a high-fat diet (HFD) for four weeks followed by a single intraperitoneal injection of streptozotocin (STZ; 40 mg/kg). Of the 40 rats, 36 developed diabetes; 32 were subsequently maintained on the HFD for an additional four weeks to establish DN and were then randomly allocated into four equal diabetic groups (*n* = 8/group) for the 8-week therapeutic intervention as follows: untreated DN, DN treated with Met alone (300 mg/kg/day), and DN treated with Met combined with either 5 or 10 mg/kg/day EDA. The diabetic animals remained on the HFD throughout the intervention period. Blood, urine, and renal tissue were collected to assess glucose homeostasis along with renal function, histopathology, oxidative stress, antioxidant defense, inflammation, apoptosis, and selected molecular regulatory markers at both gene and protein levels. DN was associated with impaired glucose homeostasis together with marked renal dysfunction, histopathological injury, oxidative stress, inflammation, and apoptosis. While Met significantly ameliorated many of these abnormalities, co-administration of EDA produced additional improvements, with the greatest overall effects observed with the higher EDA dose. Correlation and Mantel network analyses showed stronger associations of *Mapk1* expression with renal injury, inflammation, and oxidative stress markers in DN, whereas combined treatment was associated with stronger relationships between *Akt1* and *Hmox1* expression and the cytoprotective biomarkers. In conclusion, combined EDA and Met treatment produced greater overall renoprotective effects than Met monotherapy, with the higher EDA dose showing the strongest nephroprotection. Further studies are required to determine the mechanisms underlying EDA-associated renoprotection and their translational relevance in humans.

## Simple Summary

Diabetes-induced renal damage is a major complication of diabetes mellitus mediated by oxidative stress and inflammation and can progress to chronic renal failure. Hence, targeting renal oxidative stress and inflammation alongside controlling blood glucose levels could provide preventative and/or therapeutic strategies against diabetic nephropathy (DN). This study investigated whether the synthetic antioxidant and anti-inflammatory agent edaravone could enhance the protective effects of the glucose-lowering drug metformin in a rat model of DN. Compared with metformin monotherapy, combined treatment with low or high doses of edaravone produced additional improvements in insulin resistance and glycation burden, together with greater amelioration of renal oxidative stress, inflammation, apoptosis, tissue injury, and kidney dysfunction. However, co-therapy with the higher edaravone dose revealed the greatest protective effects, as indicated by the strongest renal tissue preservation along with the best modulation of molecular pathways underlying oxidative stress, inflammation, and cell survival. Together, these findings suggest that edaravone may enhance the renoprotective effects of metformin in experimental DN, with additional benefits regarding insulin resistance and glycation burden. Further studies are, however, required to precisely determine the mechanisms underlying the enhancing effects of edaravone and whether this experimental approach has potential relevance to DN in humans.

## 1. Introduction

Diabetic nephropathy (DN) is a significant complication of diabetes mellitus (DM) that can ultimately predispose patients to chronic renal disease and renal failure [1], thereby necessitating a better understanding of its associated risk factors, clinical manifestations, and management strategies [2]. DN is characterized by persistent albuminuria, a progressive decline in glomerular filtration rate (GFR), and development of hypertension, all of which contribute to increased morbidity and mortality [2,3]. The pathophysiological mechanisms underlying DN involve hyperglycemia, oxidative stress, and inflammatory processes, which collectively contribute to progressive renal impairment and an increased risk of chronic renal failure [4].

DN is a multifactorial pathology involving interconnected alterations in a variety of molecular pathways that play pivotal roles in its initiation and progression [2,5]. Chronic uncontrolled hyperglycemia triggers pathogenic metabolic alterations that promote renal injury by modulating multiple signaling pathways [1,5,6]. These intricate molecular pathways encompass oxidative stress, inflammatory signaling with cytokine dysregulation, cellular injury, autophagy, and epigenetic alterations, all of which participate in the progression of DN toward chronic renal failure [4,7]. Furthermore, the accumulation of advanced glycation end products (AGEs), increased production of reactive oxygen species (ROS), activation of growth factors and inflammatory cytokines, and stimulation of mitogen-activated protein kinase (MAPK) signaling further aggravate renal damage [8,9,10]. Additionally, increased expression of transforming growth factor-β (TGF-β), interleukin (IL)-1β, IL-6, and tumor necrosis factor-α (TNF-α), together with enhanced endoplasmic reticulum (ER) stress and activation of nuclear factor kappa-light-chain-enhancer of activated B cells (NF-κB)- and MAPK-related inflammatory pathways, also exacerbates renal inflammatory and fibrotic responses [2,8,9,10,11]. Collectively, these processes promote renal inflammation, apoptosis, glomerulosclerosis, endothelial dysfunction, and tubulointerstitial fibrosis associated with DN.

Metformin (Met), a commonly used oral hypoglycemic agent for type 2 DM, improves glycemic control and modulates several metabolic profiles, thus reducing the risk of DM-associated macrovascular complications [12]. Several studies have also reported that Met exhibits antioxidant and anti-inflammatory properties [13], which have been partly linked to inhibition of renal MAPK-associated inflammatory and fibrotic pathways [11,14]. Recent reports have further suggested that Met may slow the progression of DM-induced renal injury by attenuating pro-inflammatory mediators, including IL-1β, IL-6, TGF-β, NF-κB1, and TNF-α, reinforcing its anti-inflammatory properties [14,15,16,17,18,19,20]. Additionally, Met also reduces oxidative stress by enhancing cellular antioxidant defenses and lowering ROS generation, with several studies reporting decreased levels of the lipid peroxidation marker malondialdehyde (MDA) following treatment [21,22]. Met has also been shown to reduce albuminuria, improve GFR, and downregulate renal-cell pro-apoptotic proteins (BAX, Caspase-3), supporting a renoprotective role for Met in DN [23,24].

Edaravone (EDA) is a novel synthetic antioxidant characterized by potent scavenging activities against a variety of free radicals, including singlet oxygen, thereby limiting oxidative stress-induced cell injury [25]. Additionally, experimental studies have shown that EDA can improve endogenous antioxidant responses involving heme oxygenase-1 (HO-1), Kelch-like ECH-associated protein 1 (Keap1), and reduced glutathione (GSH), thereby alleviating neuroinflammatory injury [26,27,28]. Similarly, other studies have demonstrated potent protective anti-inflammatory effects for EDA using experimental models of ischemic, traumatic, and inflammatory neurological injury [25,29,30,31]. These neuroprotective effects have been associated with modulation of NF-κB-, nuclear factor erythroid 2-related factor 2 (Nrf2)-, and protein kinase B (Akt)-related signaling pathways, with consequent reductions in oxidative stress, inflammation, and apoptosis [27,28,32,33]. Consistent with these findings, EDA treatment has been reported to decrease levels of inflammatory mediators, including IL-1β, IL-6, and TNF-α, together with improved functional outcomes in experimental animal models of neuroinflammatory diseases [27,34,35].

Recent experiments combining Met with antioxidant and anti-inflammatory agents have demonstrated enhanced effects on oxidative stress and inflammation, supporting the potential beneficial value of adjunctive therapeutic strategies in diabetes management [13,36]. Nevertheless, evidence regarding the therapeutic effects of combined Met and EDA treatment in DN remains limited. Consequently, the present study examined whether EDA could enhance the renoprotective effects of Met in a rat model of DN by assessing glucose homeostasis, renal function, oxidative stress, inflammation, apoptosis, and histopathological injury, together with the MAPK/NF-κB1 and Akt/Nrf2 signaling pathways, to explore their potential involvement in the observed renoprotective effects of the co-treatment strategy.

## 2. Materials and Methods

### 2.1. Chemicals

Edaravone (CAS No. 89-25-8) was procured as a commercial liquid formulation (Edivone, ShinePro Life Sciences Pharmaceutical Co., Panchkula, India; 1.5 mg/20 mL ampule). Streptozotocin (STZ; CAS No. 18883-66-4) was obtained from Sigma-Aldrich (Beijing, China), whereas metformin hydrochloride (Glucophage^®^; 500 mg/tablet; CAS No. 1115-70-4) was sourced from Mina-Pharm Co. (Cairo, Egypt) under license from Merck Serono. All chemicals used in the study were of analytical grade or the highest commercially available purity.

### 2.2. Animals

In the initial phase of the study, 60 male albino Wistar rats (95 ± 10 g) were housed individually under controlled hygienic and environmental conditions at 23 ± 1 °C and 50 ± 10% relative humidity, with a 12-h light/dark cycle. The animals had unrestricted access to food and water throughout the experimental period. The study protocol was reviewed and approved by the Mansoura University Animal Care and Use Committee (MU-ACUC) prior to study commencement (13 August 2025), and the final approval certificate (No. VMR.26-07-312) was issued on 26 July 2026 following an administrative audit of the study procedures and documentation. All procedures were conducted in accordance with institutional guidelines for the care and use of laboratory animals, with appropriate measures taken to minimize animal use and distress. Male rats were selected to minimize sex-related biological heterogeneity, as gonadal hormones and sex-dependent renal responses can influence susceptibility to and progression of diabetic renal injury [37,38].

### 2.3. Induction of Type 2 Diabetes Mellitus

Sixty Wistar rats were initially randomly allocated into two experimental cohorts. The control cohort (*n* = 20) received a standard commercial rodent chow (Miladco For Poultry Forage Industry, Al-Obour City, Cairo, Egypt), comprising 50% carbohydrate, 20% protein, and 5% fat by weight, with the remaining components consisting of moisture, crude fiber, minerals/ash, and vitamins. To induce type 2 DM (T2DM), the remaining animals (*n* = 40) received a customized high-fat diet (HFD) for four weeks. The HFD was prepared by homogenizing 30% of the standard commercial chow with 53.5% unsalted butter and 11.5% pure casein powder (El-Nasr Pharmaceutical Chemicals Co.; Cairo, Egypt), supplemented with 3.5% mineral premix and 1.5% vitamin premix (El-Gomhoureya Co.; Cairo, Egypt). This formulation provided approximately 55% fat, 17.5% protein, and 15% carbohydrate by weight, and was used to promote diet-induced insulin resistance and metabolic stress [39,40,41]. Of the 20 rats maintained on the standard diet, 16 were subsequently allocated to the NC and EDA10 groups, while the remaining four were not included in the therapeutic phase and were humanely disposed of in accordance with the approved ethical protocol.

At the end of week 4, HFD-fed rats received a single intraperitoneal injection of streptozotocin (STZ; 40 mg/kg) dissolved in 1% ice-cold sodium citrate buffer (pH 4.5). This combined HFD/low-dose STZ protocol models key pathogenic features of T2DM involving HFD-induced peripheral insulin resistance followed by partial pancreatic β-cell injury rather than complete β-cell destruction [39,41]. Blood glucose levels were measured from tail-vein samples with a rapid glucometer assay 72 h after STZ injection. Rats with fasting blood glucose concentrations ≥250 mg/dL were classified as diabetic [6,40]. Of the 40 rats subjected to T2DM induction, 36 were confirmed diabetic, of which 32 were subsequently included in the DN-progression phase. The remaining four diabetic rats were not included in the subsequent experimental phase and were humanely disposed of in accordance with the approved ethical protocol. Diabetic animals remained on the HFD for an additional four weeks (weeks 5–8) to promote diabetic renal injury [6,41]. Prior to treatment, DN was considered established following 24-h urine collection in individual metabolic cages, with polyuria and proteinuria used as functional evidence of diabetic renal damage [6,20].

### 2.4. Experimental Protocols

The 16 normal and 32 diabetic rats were randomly allocated into their corresponding groups (*n* = 6 groups), each comprising eight rats. Group I (NC), serving as the negative control, received a standard rat pellet diet; Group II (EDA10) received an IP injection of EDA at 10 mg/kg/day. Groups III–VI comprised diabetic rat populations, where Group III (DN) was subjected to intragastric administration of normal saline once daily. Group IV (DN + Met) involved rats that were given oral metformin (300 mg/kg/day) in distilled water, while Group V (DN + ME5) and Group VI (DN + ME10) respectively included rats treated with IP EDA at a low (5 mg/kg) and high (10 mg/kg) dose in combination with oral Met at 300 mg/kg/day, for a continuous eight-week period (weeks 9–16). The dosages of Met and EDA were established based on investigations conducted by [20] and [42], respectively. The overall experimental design and animal allocation are summarized in Supplementary Figure S1.

### 2.5. Sample Collection

Sample collection was performed 48 h after the final treatment dose at the end of the 8-week therapeutic intervention (week 16). Twenty-four-hour urine samples were collected from individual rats using a metabolic cage. The animals were then sedated by ketamine (50 mg/kg) and xylazine (10 mg/kg) intraperitoneal injection, as previously described [43,44]. Blood samples were then collected from the retro-orbital plexus, after which the rats were euthanized by cervical dislocation. Blood samples were centrifuged at 1500× *g* for 10 min, and the supernatant was stored at −30 °C until biochemical analysis [43].

Both kidneys were harvested from each rat. A 100-mg renal tissue specimen was homogenized in phosphate-buffered saline (PBS; pH 7.4) at a 1:10 (w/v) tissue-to-buffer ratio and centrifuged to obtain the supernatant for measurement of cytokines, oxidative stress and antioxidant biomarkers, and renal injury markers. Additionally, another kidney specimen was preserved in 10% neutral buffered formaldehyde for histological studies, whereas the remaining tissues were cryopreserved in liquid nitrogen for quantitative gene expression by PCR.

### 2.6. Urine Biochemical Analysis

Twenty-four-hour urine volumes were recorded. Urinary total protein and creatinine (Cr) concentrations were then measured using an automated chemistry analyzer (Mindray BS240, Nanjing, China). Cr clearance was calculated using the standard formula, whereas urinary protein excretion was normalized to urinary Cr and is expressed as the protein-to-Cr ratio, calculated as urinary total protein (mg) divided by urinary Cr (mg), as previously reported [43,44]. β2-microglobulin urine concentrations were quantified using a rat-specific ELISA kit (#ab264619; Abcam, Cambridge, UK) according to the manufacturer’s instructions.

### 2.7. Blood Biochemical Profiles

Fasting blood glucose was measured before sedation and euthanasia using the same glucometer-based method described above. Glycated hemoglobin (HbA1c; #MBS2033689; MyBioSource, San Diego, CA, USA), fasting insulin (#E-EL-R3034; Elabscience, Houston, TX, USA), cystatin C (#E-EL-R0304; Elabscience), and AGEs (#ELK1890; ELK Biotechnology Co., Ltd., Sugar Land, TX, USA) were measured using rat-specific ELISA kits according to the manufacturers’ instructions. Serum creatinine, urea, uric acid (UA), total protein, and albumin were measured using an automated chemistry analyzer (Mindray BS240) according to the corresponding reagent protocols. Insulin resistance was estimated using the homeostatic model assessment of insulin resistance (HOMA-IR) [45], as follows:

$$\text{HOMA-IR}=\left[\frac{\mathrm{Fasting}\;\mathrm{glucose}\;(\mathrm{mg}/\mathrm{dL})\times \mathrm{Fasting}\;\mathrm{insulin}\;(µ\mathrm{IU}/\mathrm{mL})}{405}\right]$$

### 2.8. ELISA Analysis of Renal Inflammatory, Oxidative Stress, Antioxidant, Injury, and Apoptosis Biomarkers

Rat-specific ELISA kits from Elabscience were used to quantify renal tissue concentrations of IL-1β (#E-EL-R0012), IL-6 (#E-EL-R0015), interleukin-10 (IL-10; #E-EL-R0016), TNF-α (#E-EL-R2856), TGF-β1 (#E-UNEL-R0054), kidney injury molecule-1 (KIM-1; #E-EL-R3019), neutrophil gelatinase-associated lipocalin (NGAL; #E-EL-R3055), and MDA (#E-EL-0060).

TLR4 (#MBS705488), NF-κB (#MBS287521), Nrf2 (#MBS012148), inducible nitric oxide synthase (iNOS; #MBS263618), protein carbonyl (#MBS2600784), BAX (#MBS2512405), Caspase-3 (#MBS261814), and B-cell lymphoma 2 (BCL-2; #MBS2515143) were quantified using rat-specific assay kits from MyBioSource.

Similarly, GSH (#MBS732529), catalase (CAT; #MBS701908), superoxide dismutase (SOD; #MBS036924), and glutathione peroxidase 1 (GPx1; #MBS3809062) were measured using rat-specific ELISA kits from MyBioSource, whereas 8-hydroxy-2′-deoxyguanosine (8-OHdG; #ELK10848) was measured using a rat-specific ELISA kit from ELK Biotechnology Co., Ltd. All samples were assayed in duplicate according to the manufacturers’ instructions using a fully automated ELISA analyzer (Elisys Uno, Human, Wiesbaden, Germany).

### 2.9. Renal Gene Expression

Total RNA was extracted from 30 mg of renal tissue using a total RNA extraction kit (#R1200; Solarbio Science & Technology Co., Ltd., Beijing, China) according to the manufacturer’s instructions. RNA concentration and purity were assessed using a NanoDrop One spectrophotometer (Thermo Fisher Scientific, Waltham, MA, USA). The extracted RNA was reverse-transcribed into complementary DNA (cDNA) using the BeyoRT™ III First Strand cDNA Synthesis Kit (#D7178M; Beyotime Biotechnology Co., Ltd., Shanghai, China) according to the manufacturer’s protocol.

Quantitative real-time PCR was performed using a SYBR Green PCR kit (#204343; Qiagen, Hilden, Germany), as previously described [44]. Amplification was performed for 40 cycles, with each cycle consisting of denaturation at 95 °C for 15 s followed by annealing/extension at 60 °C for 1 min. Two negative controls were included: a no-template control in which cDNA was replaced with nuclease-free water, and a no-reverse-transcription control containing total RNA that had not undergone reverse transcription. Each reaction was performed in triplicate in a final volume of 20 µL containing 10 µL SYBR Green master mix, 7 µL nuclease-free DEPC-treated water, 1 µL of each primer (5 pmol), and 1 µL cDNA containing 25 ng of template.

Both GAPDH and β-actin (*Actb*) were evaluated as candidate internal control genes, with β-actin showing stable expression across the experimental groups and therefore being selected as the internal reference gene for normalization. GAPDH was not used for normalization of the reported target-gene expression data. The expression of *Mapk1*, *Akt1*, *Keap1*, and *Hmox1* was analyzed using *Actb* for normalization. All primer sequences are listed in Supplementary Table S1. Relative expression of the target genes was calculated using the 2^−ΔΔCt^ method, as described earlier [46].

### 2.10. Histopathological Studies

Kidney specimens were fixed in 10% neutral buffered formaldehyde, dehydrated through graded ethanol, cleared in xylene, embedded in paraffin, sectioned at 2 µm thickness, and stained with hematoxylin and eosin (H&E). Renal sections were examined microscopically at 200× and 400× magnification.

Histopathological assessment was performed blindly in five randomly selected, non-overlapping fields from each renal section. Tubular atrophy was semiquantitatively scored according to the percentage of the affected renal area as follows—0, absent; 1, <25%; 2, 25–50%; and 3, >50%—according to previously described criteria [47]. Interstitial edema was graded semiquantitatively according to lesion severity as follows—0, absent; 1, mild; 2, moderate; and 3, severe—using a modified version of a previously described renal histopathological scoring approach [48].

### 2.11. Statistical Analysis

Data analysis and visualization were performed using R (version 4.3.1; R Foundation for Statistical Computing, Vienna, Austria) executed within the RStudio (version 4.3.1; 2023-06-16, Build 524). integrated development environment (Posit; Boston, MA, USA). Data normality was assessed using the Shapiro–Wilk and Kolmogorov–Smirnov tests. No significant departures from normality were detected (*p* > 0.05); therefore, results are shown as mean ± standard deviation (SD). Differences among experimental groups were analyzed using one-way ANOVA, followed by Tukey’s multiple-comparison post hoc test. Statistical significance was defined as *p* < 0.05. Pairwise significance values presented in the tables and figures represent Tukey-adjusted *p* values.

For heatmap visualization, normalized gene expression values were transformed to z-scores to facilitate comparisons among genes with different expression patterns. Hierarchical clustering of biological replicates and variables was performed using Euclidean distance and complete-linkage clustering. Pairwise associations among the evaluated renal injury biomarkers and histopathological indices were assessed using Pearson correlation coefficients (r). Associations between gene expression profiles and the corresponding matrices of renal tissue injury biomarkers were evaluated using Mantel tests. The Mantel correlation coefficient (Mantel’s r) and associated *p* values were calculated using the vegan package.

Bar plots were generated using ggplot2 (version 3.5.1), whereas hierarchical clustering heatmaps were generated using ComplexHeatmap (version 2.15.4) and circlize. Lower-triangular Pearson correlation heatmaps were generated using the qcorrplot function, and Mantel correlation networks were visualized using linkET (version 0.1.0), together with ggplot2, vegan, and patchwork (version 1.2.0).

## 3. Results

### 3.1. Glycation Burden and Insulin Resistance

As shown in Table 1, there were no significant differences in fasting blood glucose, HbA1c, fasting insulin, HOMA-IR, or serum AGEs between the NC and EDA10 groups (*p* > 0.05). In contrast, the DN group exhibited marked increases in fasting glucose, HbA1c, fasting insulin, HOMA-IR, and serum AGEs compared with both the NC and EDA10 groups (*p* ≤ 0.001), consistent with pronounced hyperglycemia, insulin resistance, and increased glycation burden. Although Met treatment significantly reduced all five parameters compared with untreated DN rats (*p* ≤ 0.001), their values remained significantly higher than those in the NC group.

Co-administration of low and high doses of EDA with Met produced parameter-specific effects. Although fasting glucose, HbA1c, and fasting insulin concentrations were numerically lower in both co-treatment groups than in the DN + Met group, these differences were not statistically significant (*p* > 0.05). In contrast, HOMA-IR was significantly lower in both DN + ME5 and DN + ME10 than in DN + Met (*p* ≤ 0.01). However, HOMA-IR remained significantly higher than NC in the DN + ME5 group (*p* < 0.05), whereas the DN + ME10 group did not differ significantly from NC (*p* > 0.05).

Serum AGEs also showed significant additional reductions with combined treatment. Compared with DN + Met, AGEs were significantly lower in both DN + ME5 (*p* ≤ 0.01) and DN + ME10 (*p* ≤ 0.001). The DN + ME5 group remained significantly different from NC (*p* < 0.05), whereas serum AGEs in DN + ME10 did not differ significantly from NC (*p* > 0.05). No significant differences were detected between DN + ME5 and DN + ME10 for any of the parameters evaluated (*p* > 0.05).

### 3.2. Combined Edaravone and Metformin Therapy Mitigates Renal Dysfunction in Rats with Diabetic Nephropathy

**Figure 1:**
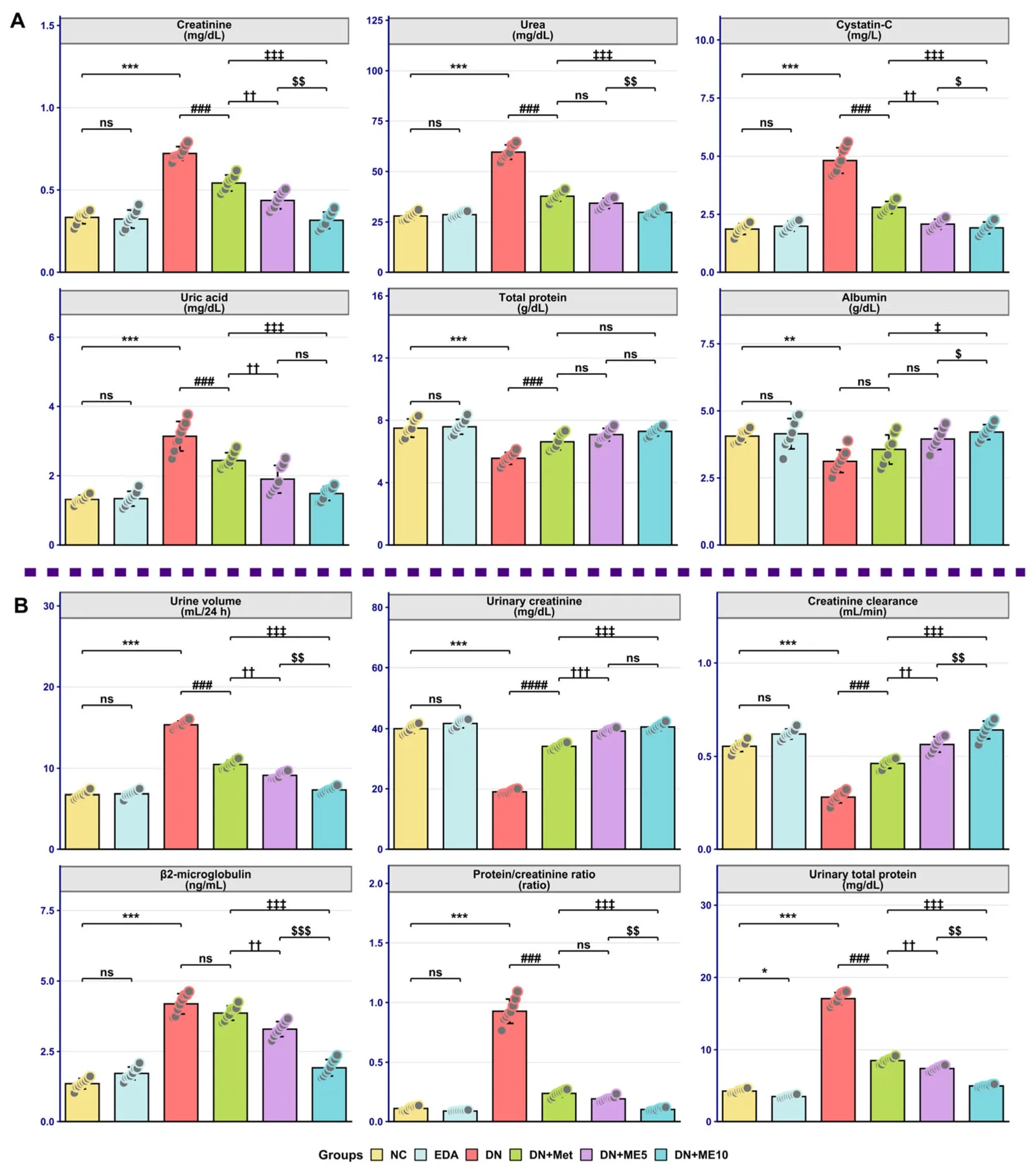
**Effects of metformin alone and in combination with edaravone on renal function and urinary injury indices in rats with diabetic nephropathy.** (**A**) Serum renal-function biomarkers. (**B**) Twenty-four-hour urinary renal-function and injury biomarkers. Data are presented as mean ± SD (*n* = 8 rats/group). Colored bars represent group means, whereas individual dots represent biological replicates. Differences among groups were analyzed using one-way ANOVA followed by Tukey’s multiple-comparison post hoc test. Tukey-adjusted *p* values for the indicated pairwise comparisons are shown by brackets. Asterisks denote comparisons with NC (* *p* < 0.05, ** *p* ≤ 0.01, *** *p* ≤ 0.001); hash symbols denote DN versus DN + Met (### *p* ≤ 0.001, #### *p* ≤ 0.0001); daggers denote DN + Met versus DN + ME5 (†† *p* ≤ 0.01, ††† *p* ≤ 0.001); double daggers denote DN + Met versus DN + ME10 (‡ *p* < 0.05, ‡‡‡ *p* ≤ 0.001); and dollar signs denote DN + ME5 versus DN + ME10 ($ *p* < 0.05, $$ *p* ≤ 0.01, $$$ *p* ≤ 0.001). Non-significant comparisons are indicated by ns (*p* > 0.05). NC, negative control; EDA10, non-diabetic control treated with edaravone (10 mg/kg); DN, untreated diabetic nephropathy; DN + Met, diabetic nephropathy treated with metformin (300 mg/kg); DN + ME5, diabetic nephropathy treated with metformin plus edaravone (5 mg/kg); DN + ME10, diabetic nephropathy treated with metformin plus edaravone (10 mg/kg).

As demonstrated in Figure 1, no significant differences were observed between the NC and EDA10 groups in any of the serum or urinary biomarkers of renal function (*p* > 0.05). In contrast, the DN group exhibited marked renal dysfunction, with significant increases in serum creatinine, urea, cystatin C, and uric acid, together with reductions in total protein and albumin compared with the NC group (*p* < 0.0001; Figure 1A). Furthermore, the DN group showed significant increases in 24-h urine volume, total urinary protein excretion, urinary β2-microglobulin, and the protein-to-creatinine ratio, together with reductions in urinary creatinine and creatinine clearance compared with the NC group (*p* < 0.0001; Figure 1B). Met monotherapy significantly improved most of these abnormalities relative to untreated DN rats, with lower serum creatinine, urea, cystatin C, and uric acid concentrations and higher total protein levels (*p* < 0.01; Figure 1A). Similarly, the DN + Met group showed significant improvements in most urinary indices of renal dysfunction relative to untreated DN rats, except for urinary β2-microglobulin (*p* < 0.01; Figure 1B).

Co-administration of EDA with Met produced additional improvements in renal function relative to Met monotherapy (Figure 1). The DN + ME5 group generally showed intermediate improvements in serum and urinary renal indices relative to DN + Met, although statistical significance varied among individual parameters. The higher-dose combination produced more pronounced changes compared with DN + Met, with significantly lower serum creatinine, urea, cystatin C, and uric acid concentrations and higher albumin levels, whereas total protein did not differ significantly (Figure 1A). Similarly, DN + ME10 showed significant improvements in 24-h urine volume, urinary creatinine, creatinine clearance, urinary β2-microglobulin, total urinary protein excretion, and the protein-to-creatinine ratio compared with DN + Met (Figure 1B). Several of these urinary indices were also significantly improved in DN + ME10 compared with DN + ME5 (*p* ≤ 0.01), indicating a greater improvement in renal function with the higher EDA dose.

### 3.3. Effects of Metformin and Edaravone on Renal Inflammatory, Oxidative-Stress, and Antioxidant Markers

As shown in Table 2, none of the measured renal inflammatory, oxidative-stress, or antioxidant markers differed significantly between the NC and EDA10 groups (*p* > 0.05). In contrast, DN was associated with substantial elevations in TNF-α, IL-1β, IL-6, TLR4, NF-κB1, iNOS, protein carbonyl, MDA, and 8-OHdG, together with a reduction in IL-10, Nrf2, GSH, GPx1, CAT, and SOD compared with NC (*p* ≤ 0.001 for all).

Met monotherapy significantly reduced TNF-α, IL-1β, IL-6, TLR4, NF-κB1, iNOS, protein carbonyl, MDA, and 8-OHdG and increased IL-10, Nrf2, GSH, CAT, and SOD relative to untreated DN rats, whereas GPx1 did not differ significantly between the two groups (Table 2). Despite these improvements, TNF-α, IL-1β, IL-6, TLR4, NF-κB1, Nrf2, GPx1, CAT, SOD, iNOS, protein carbonyl, MDA, and 8-OHdG remained significantly different from NC.

Addition of EDA to Met produced further parameter-specific improvements. Compared with DN + Met, DN + ME5 showed significant reductions in TNF-α, IL-1β, IL-6, TLR4, NF-κB1, iNOS, and 8-OHdG and significant increases in IL-10, Nrf2, GPx1, CAT, and SOD, whereas GSH, protein carbonyl, and MDA did not differ significantly. Similarly, DN + ME10 showed significant improvements in TNF-α, IL-1β, IL-6, TLR4, NF-κB1, IL-10, Nrf2, GPx1, CAT, SOD, iNOS, protein carbonyl, and 8-OHdG compared with DN + Met, whereas GSH and MDA were not significantly different between these groups. In the DN + ME10 group, TNF-α, IL-1β, IL-6, TLR4, NF-κB1, IL-10, Nrf2, GSH, GPx1, CAT, SOD, protein carbonyl, MDA, and 8-OHdG did not differ significantly from NC, whereas iNOS remained elevated (*p* < 0.05). Direct comparison of the two co-treatment groups showed significantly lower TNF-α, IL-1β, iNOS, protein carbonyl, and 8-OHdG in DN + ME10 than in DN + ME5, while the remaining markers did not differ significantly.

### 3.4. Effects of Metformin and Edaravone on Renal Injury, Profibrotic, and Apoptotic Markers

**Figure 2:**
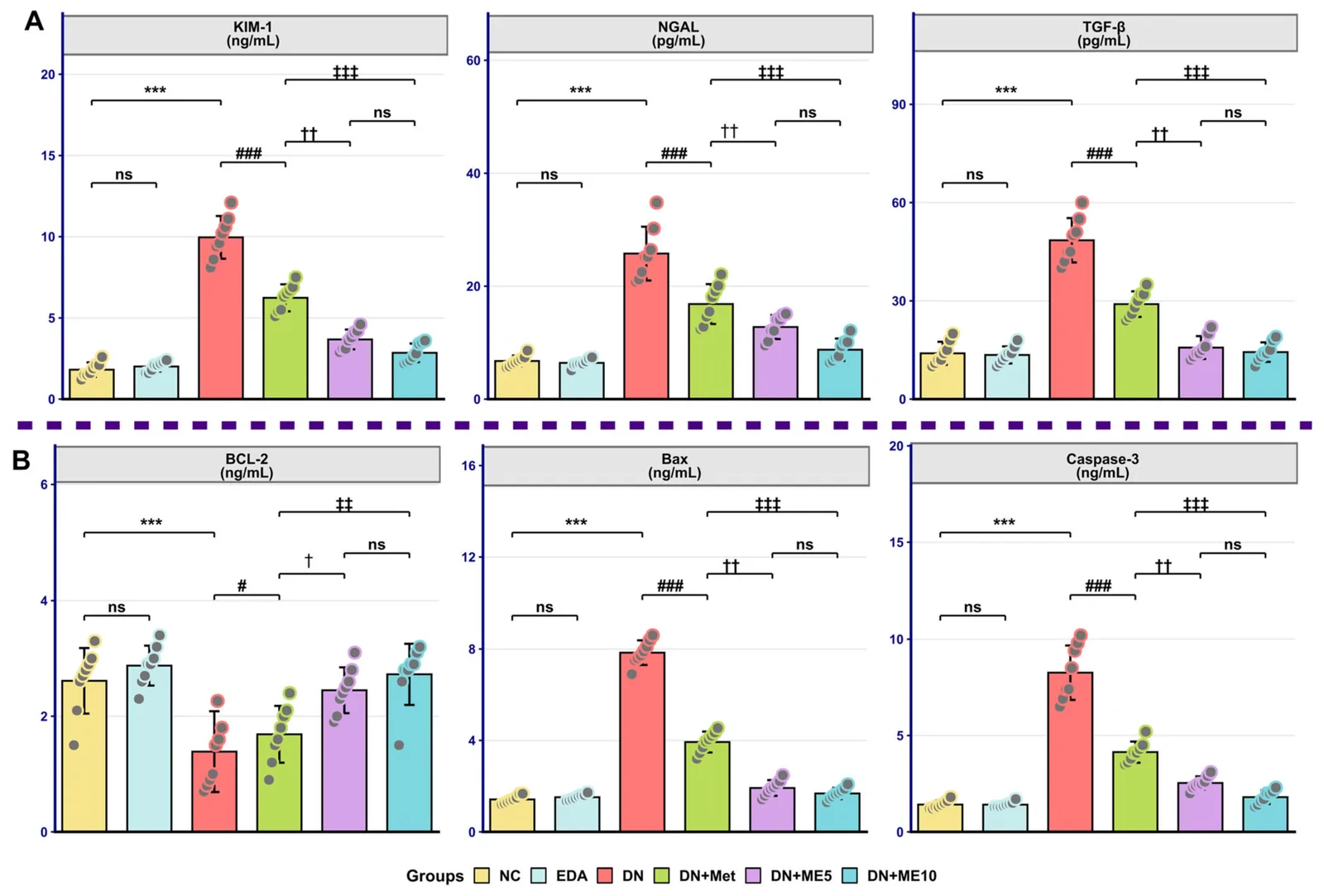
**Effects of metformin alone and in combination with edaravone on renal injury, profibrotic, and apoptotic markers in rats with diabetic nephropathy.** (**A**) Renal injury and fibrosis biomarkers. (**B**) Apoptotic and anti-apoptotic markers. Data are presented as mean ± SD (*n* = 8 rats/group). Colored bars represent group means, whereas individual dots represent biological replicates. Differences among groups were analyzed using one-way ANOVA followed by Tukey’s multiple-comparison post hoc test. Tukey-adjusted *p* values for the indicated pairwise comparisons are shown by brackets. Asterisks denote comparisons with NC (*** *p* ≤ 0.001); hash symbols denote DN versus DN + Met (# *p* < 0.05, ### *p* ≤ 0.001); daggers denote DN + Met versus DN + ME5 († *p* < 0.05, †† *p* ≤ 0.01); and double daggers denote DN + Met versus DN + ME10 (‡‡ *p* ≤ 0.01, ‡‡‡ *p* ≤ 0.001). Non-significant comparisons are indicated by ns (*p* > 0.05). NC, negative control; EDA10, non-diabetic control treated with edaravone (10 mg/kg); DN, untreated diabetic nephropathy; DN + Met, diabetic nephropathy treated with metformin (300 mg/kg); DN + ME5, diabetic nephropathy treated with metformin plus edaravone (5 mg/kg); DN + ME10, diabetic nephropathy treated with metformin plus edaravone (10 mg/kg).

As shown in Figure 2, none of the measured renal injury, profibrotic, or apoptotic markers differed significantly between the NC and EDA10 groups (*p* > 0.05). In contrast, DN was associated with significant increases in renal KIM-1, NGAL, and TGF-β1 concentrations (Figure 2A), together with increased BAX and Caspase-3 and reduced BCL-2 compared with NC (*p* ≤ 0.001; Figure 2B).

Met monotherapy significantly reduced KIM-1, NGAL, TGF-β1, BAX, and Caspase-3 and increased BCL-2 relative to untreated DN rats (*p* < 0.01). However, all six markers remained significantly different from NC, indicating incomplete recovery with Met alone.

Combining EDA with Met was associated with better improvements in all six markers. Compared with DN + Met, DN + ME5 showed significantly lower KIM-1, NGAL, TGF-β1, BAX, and Caspase-3 and higher BCL-2. KIM-1, NGAL, and Caspase-3; nevertheless, these remained significantly different from NC in the DN + ME5 group, whereas TGF-β1, BAX, and BCL-2 were comparable between both groups. While the DN + ME10 group also showed significant alleviations in all six markers compared with DN + Met, none differed significantly from NC. Direct comparison between DN + ME5 and DN + ME10 revealed no significant differences in any of the measured markers (*p* > 0.05).

### 3.5. Effects of Metformin and Edaravone on Renal Mapk1, Akt1, Keap1, and Hmox1 Expression

**Figure 3:**
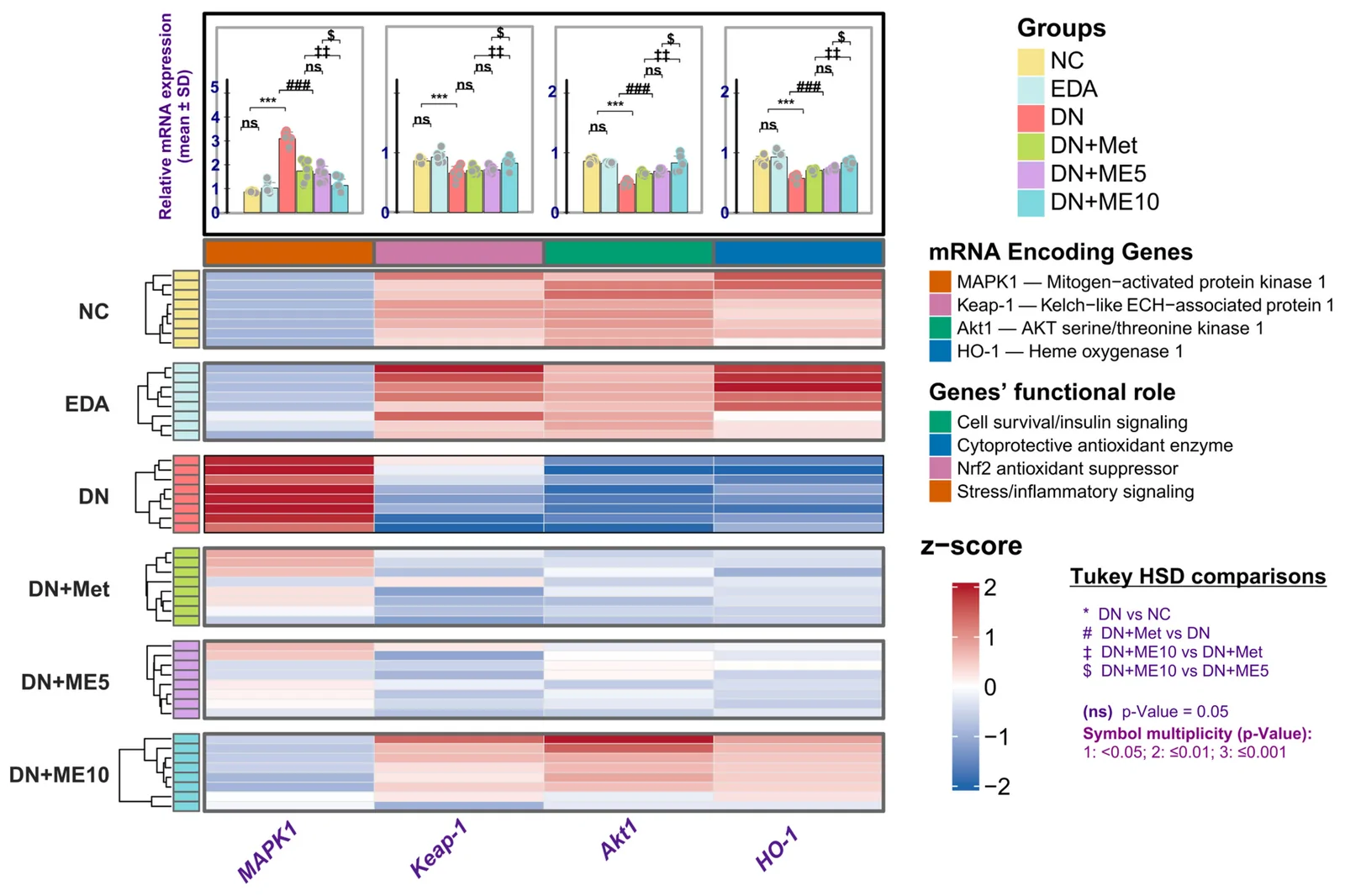
**Integrated complex heatmap and expression profiling of mRNA encoding genes signaling renal redox status and cell survival cascades in rats with diabetic nephropathy.** The upper panels show relative mRNA expression of *Mapk1*, *Keap1*, *Akt1*, and *Hmox1*, presented as mean ± SD (*n* = 8 rats/group), with individual dots representing biological replicates. Differences among groups were analyzed using one-way ANOVA followed by Tukey’s multiple-comparison post hoc test. Tukey-adjusted *p* values for the indicated pairwise comparisons are shown by brackets. Asterisks denote comparisons with NC (*** *p* ≤ 0.001); hash symbols denote DN versus DN + Met (### *p* ≤ 0.001); double daggers denote DN + Met versus DN + ME10 (‡‡ *p* ≤ 0.01); and dollar signs denote DN + ME5 versus DN + ME10 ($ *p* < 0.05). Non-significant comparisons are indicated by ns (*p* > 0.05). The colored annotation strip identifies the principal biological role assigned to each analyzed gene: *Mapk1*, stress-responsive/inflammatory signaling; *Keap1*, negative regulation of Nrf2 signaling; *Akt1*, cell-survival/insulin signaling; and *Hmox1*, cytoprotective antioxidant activity. The lower heatmap shows z-score-transformed relative expression values for individual biological replicates within each experimental group, with red indicating higher and blue indicating lower relative expression. Within each experimental group, biological replicates were hierarchically clustered using Euclidean distance and complete-linkage clustering. NC, negative control; EDA10, non-diabetic control treated with edaravone (10 mg/kg); DN, untreated diabetic nephropathy; DN + Met, diabetic nephropathy treated with metformin (300 mg/kg); DN + ME5, diabetic nephropathy treated with metformin plus edaravone (5 mg/kg); DN + ME10, diabetic nephropathy treated with metformin plus edaravone (10 mg/kg).

Renal expression of *Mapk1*, *Akt1*, *Keap1*, and *Hmox1* differed significantly among the experimental groups (Figure 3). While no significant differences were observed between the NC and EDA10 groups for any of the four genes, DN was associated with marked upregulation of *Mapk1* and significant downregulation of *Akt1*, *Keap1*, and *Hmox1* relative to the NC group.

Met treatment partially reversed these transcriptional alterations. Relative to untreated DN rats, DN + Met showed significantly lower *Mapk1* expression and higher *Akt1* and *Hmox1* expression, whereas *Keap1* did not differ significantly between the two groups. Nevertheless, all four genes remained significantly different from NC following Met monotherapy (Figure 3).

While the DN + ME5 group did not differ significantly from DN + Met for any of the four genes, DN + ME10 showed lower *Mapk1* and higher *Akt1*, *Keap1*, and *Hmox1* expression than DN + Met. None of the four genes differed significantly between DN + ME10 and NC. Direct comparison of the two co-treatment groups also showed significant differences in all four genes, with DN + ME10 producing a greater shift toward the expression pattern observed in the non-diabetic controls (Figure 3).

### 3.6. Histopathological Results

**Figure 4:**
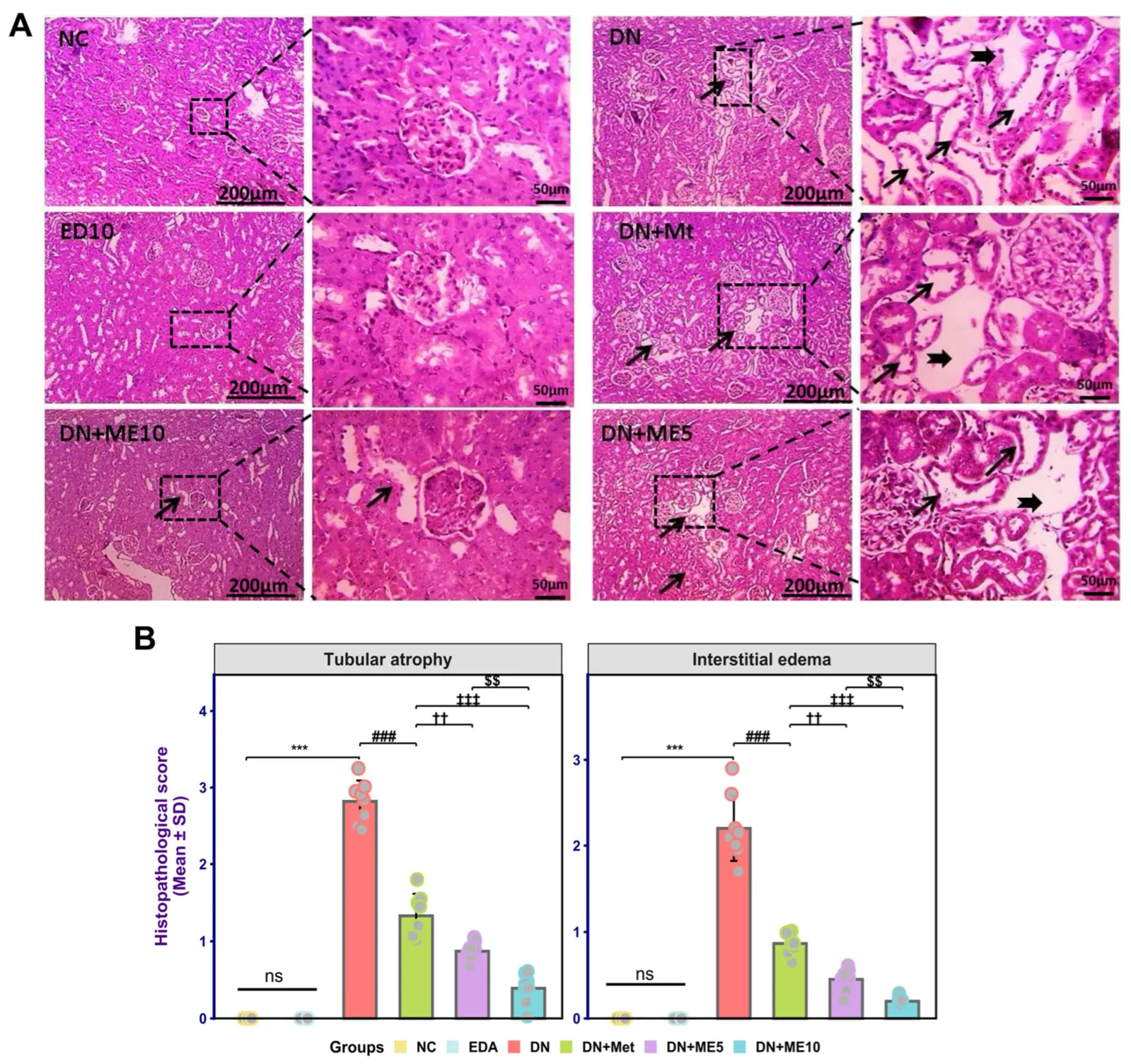
**Representative H&E-stained renal sections and semiquantitative histopathological lesion scores.** (**A**) The NC and EDA10 groups showed preserved glomerular and tubular architecture with minimal interstitial changes. The DN group exhibited marked tubular dilation and atrophy (thin arrows), accompanied by interstitial edema (thick arrows). These alterations were less pronounced in the DN + Met and DN + ME5 groups, whereas the DN + ME10 group showed only slight tubular dilation and atrophy. Images were obtained at 200× magnification (scale bar = 200 µm) and 400× magnification (scale bar = 50 µm). (**B**) Semiquantitative scores for tubular atrophy and interstitial edema. Data are presented as mean ± SD (*n* = 8 rats/group). Colored bars represent group means, whereas individual dots represent biological replicates. Differences among groups were analyzed using one-way ANOVA followed by Tukey’s multiple-comparison post hoc test. Tukey-adjusted *p* values for the indicated pairwise comparisons are shown by brackets. Asterisks denote comparisons with NC (*** *p* ≤ 0.001); hash symbols denote DN versus DN + Met (### *p* ≤ 0.001); daggers denote DN + Met versus DN + ME5 (†† *p* ≤ 0.01); double daggers denote DN + Met versus DN + ME10 (‡‡‡ *p* ≤ 0.001); and dollar signs denote DN + ME5 versus DN + ME10 ($$ *p* ≤ 0.01). Non-significant comparisons are indicated by ns (*p* > 0.05). NC, negative control; EDA10, non-diabetic control treated with edaravone (10 mg/kg); DN, untreated diabetic nephropathy; DN + Met, diabetic nephropathy treated with metformin (300 mg/kg); DN + ME5, diabetic nephropathy treated with metformin plus edaravone (5 mg/kg); DN + ME10, diabetic nephropathy treated with metformin plus edaravone (10 mg/kg).

Microscopic examination of H&E-stained renal sections showed preserved glomerular and tubular architecture with minimal interstitial changes in the NC and EDA10 groups (Figure 4A). In contrast, kidneys from untreated DN rats showed prominent tubular dilation and atrophy, interstitial edema and inflammatory infiltration, together with vacuolar degeneration and cytoplasmic swelling of tubular epithelial cells.

Met monotherapy reduced the severity of these structural abnormalities. Sections from the DN + Met group showed moderate tubular dilation and atrophy with persistent interstitial edema, whereas the DN + ME5 group displayed milder tubular and interstitial changes. The DN + ME10 group showed the greatest preservation of renal morphology, with largely maintained glomerular architecture, only slight tubular dilation and atrophy, and less prominent tubular epithelial degeneration. Mild residual vacuolar degeneration remained detectable in some tubular epithelial cells (Figure 4A).

Semiquantitative analysis aligned with the microscopic observations. Tubular atrophy and interstitial edema scores were significantly higher in DN than in NC (*p* ≤ 0.001; Figure 4B). Both scores were significantly lower in DN + Met than in untreated DN rats (*p* ≤ 0.001) and were further reduced in DN + ME5 compared with DN + Met (*p* ≤ 0.01). DN + ME10 showed additional reductions in both tubular atrophy and interstitial edema scores compared with DN + Met (*p* ≤ 0.001) and DN + ME5 (*p* ≤ 0.01), indicating greater histopathological improvement with the higher EDA dose.

### 3.7. Correlation and Mantel Network Analysis of Renal Injury, Inflammatory, Oxidative-Stress, and Gene-Expression Profiles

#### 3.7.1. Associations in Untreated DN and Metformin-Treated Rats

**Figure 5:**
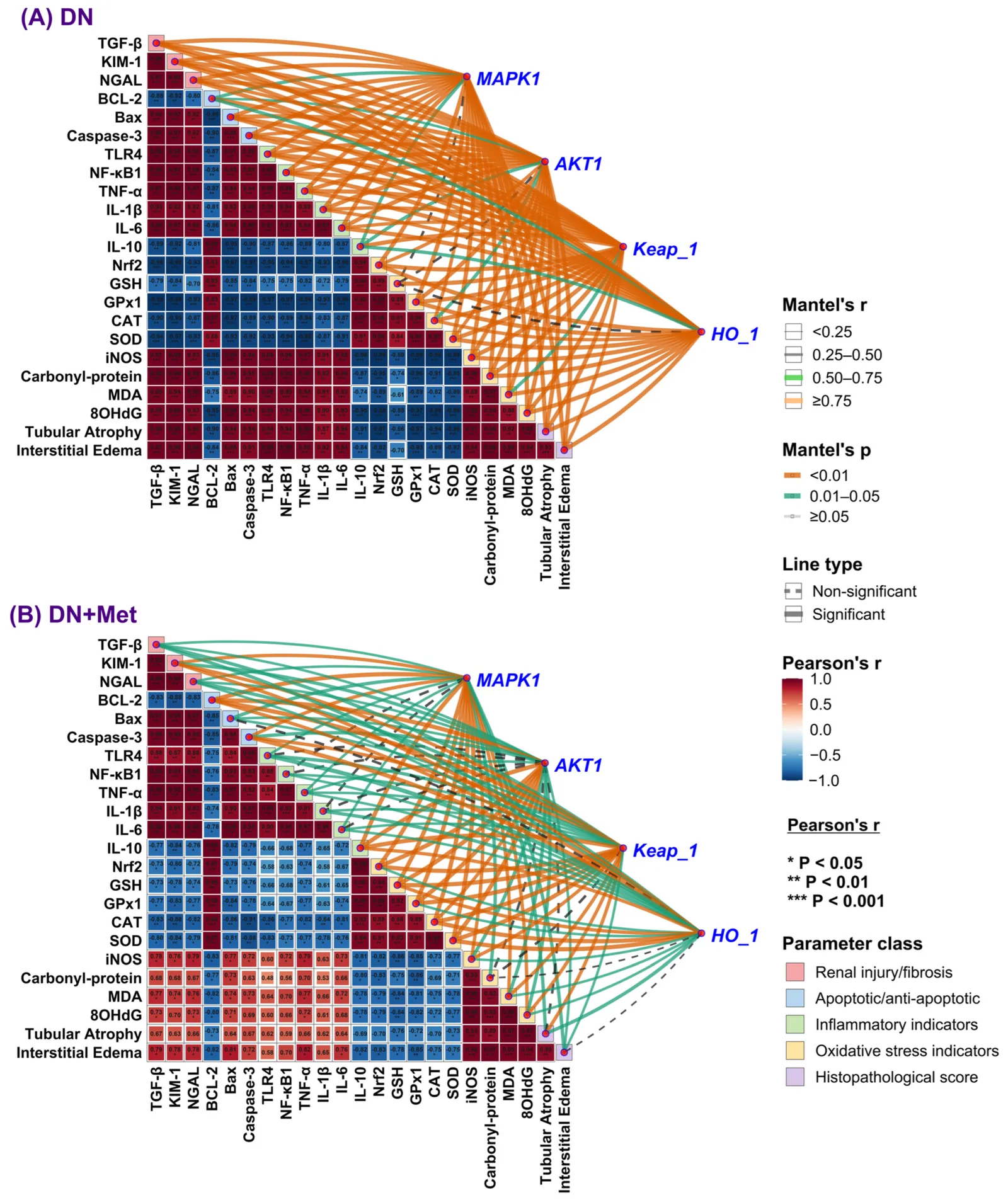
**Pearson correlation matrices and Mantel network analysis of renal biochemical, histopathological, and gene expression variables in untreated and metformin-treated diabetic nephropathy.** (**A**) Untreated diabetic nephropathy (DN) group. (**B**) Metformin-treated diabetic nephropathy (DN + Met) group. Lower-triangular heatmaps show Pearson correlation coefficients (*r*), with red indicating positive and blue indicating negative correlations. Mantel network links show associations between renal expression of *Mapk1*, *Akt1*, *Keap1*, and *Hmox1* and the corresponding biomarker clusters. Line thickness represents Mantel’s *r*, whereas line color indicates the associated Mantel *p*-value. Solid and dashed lines distinguish significant and non-significant Mantel associations, respectively. Pearson correlations and Mantel associations were considered statistically significant at *p* < 0.05. * *p* < 0.05, ** *p* < 0.01, *** *p* < 0.001.

In the DN group (Figure 5A), Pearson correlation analysis showed strong positive correlations among renal injury and profibrotic markers (TGF-β1, KIM-1, and NGAL), inflammatory mediators (TLR4, NF-κB1, TNF-α, IL-1β, and IL-6), oxidative-stress markers (iNOS, protein carbonyl, MDA, and 8-OHdG), pro-apoptotic markers (BAX and Caspase-3), and histopathological scores for tubular atrophy and interstitial edema. Most of these correlations were very strong (*r* = 0.85–0.99, *p* < 0.05 to ≤ 0.01). Conversely, Nrf2, GSH, GPx1, CAT, SOD, BCL-2, and IL-10 showed strong inverse correlations with indices of renal tissue injury (*r* = −0.70 to −0.97, *p* < 0.05 to ≤ 0.01).

Mantel analysis identified particularly strong associations between *Mapk1* expression and clusters of renal injury, inflammation, oxidative stress, and histopathological damage (Mantel’s *r* ≥ 0.75, *p* ≤ 0.01). In comparison, *Akt1* and *Hmox1* showed fewer significant associations with these biomarker clusters in untreated DN rats.

Following Met treatment (Figure 5B), the strength of many positive correlations among renal injury, inflammatory, and oxidative-stress markers was reduced, shifting from predominantly very strong correlations in untreated DN to moderate-to-strong relationships (*r* ≈ 0.60–0.85). Antioxidant markers retained inverse correlations with pathological indices. Mantel analysis also showed weaker associations of *Mapk1* with injury-related variables and more prominent associations of *Akt1* and *Hmox1* with markers related to antioxidant and tissue-protective responses (Mantel’s *r* ≥ 0.60, *p* < 0.05).

#### 3.7.2. Correlation Network Remodeling Following Co-Administration with Edaravone

**Figure 6:**
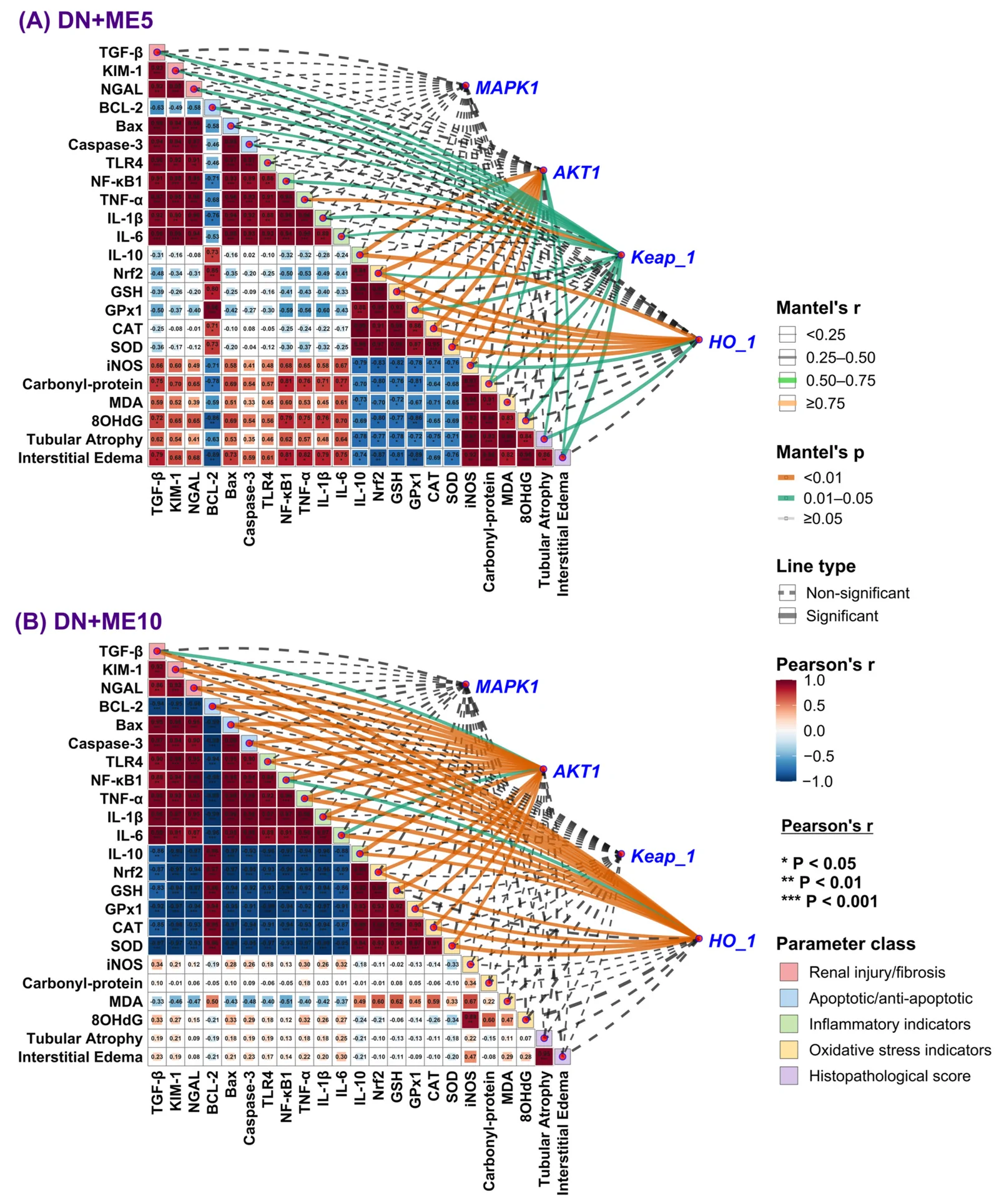
**Pearson correlation matrices and Mantel network analysis of renal biochemical, histopathological, and gene-expression variables following combined edaravone and metformin treatment.** (**A**) DN + ME5 group treated with metformin plus edaravone (5 mg/kg). (**B**) DN + ME10 group treated with metformin plus edaravone (10 mg/kg). Lower-triangular heatmaps show Pearson correlation coefficients (*r*), with red indicating positive and blue indicating negative correlations. Mantel network links show associations between renal expression of *Mapk1*, *Akt1*, *Keap1*, and *Hmox1* and the corresponding biomarker clusters. Line thickness represents Mantel’s *r*, whereas line color indicates the associated Mantel *p* value. Solid and dashed lines distinguish significant and non-significant Mantel associations, respectively. Pearson correlations and Mantel associations were considered statistically significant at *p* < 0.05.

In the DN + ME5 group (Figure 6A), Pearson analysis showed weaker positive correlations among renal injury, inflammatory, and oxidative-stress markers than those observed in the DN and DN + Met groups. Conversely, Nrf2, GSH, GPx1, CAT, SOD, IL-10, and BCL-2 showed strong positive correlations with one another (*r* = 0.70–0.95, *p* < 0.05 to ≤ 0.01) and moderate-to-strong inverse correlations with renal injury, inflammatory, oxidative-stress, and histopathological indices (*r* = −0.60 to −0.90, *p* < 0.05 to ≤ 0.01).

Mantel analysis in DN + ME5 showed several significant associations of *Akt1* and *Hmox1* expression with antioxidant and anti-inflammatory markers (Mantel’s *r* > 0.50, *p* < 0.05), whereas associations involving *Mapk1* and injury-related variables were less prominent than in untreated DN rats.

In the DN + ME10 group (Figure 6B), Pearson analysis showed a clearer separation between protective and pathological biomarker patterns. Antioxidant, anti-inflammatory, and anti-apoptotic markers were strongly directly correlated with one another (*r* ≈ 0.80–0.99, *p* < 0.05), while showing predominantly strong inverse correlations with renal injury, inflammatory, oxidative-stress, and histopathological indices. Mantel analysis identified numerous significant associations involving *Hmox1* and *Akt1* with protective biomarker clusters (Mantel’s *r* frequently > 0.50, *p* < 0.05), whereas most associations involving *Keap1* were weak and non-significant (*p* > 0.05).

Compared with DN + ME5, the DN + ME10 group showed a more distinct correlation pattern characterized by stronger associations of *Akt1* and *Hmox1* with protective biomarkers and weaker relationships between these protective variables and indices of renal injury.

## 4. Discussion

The present study investigated whether EDA could enhance the renoprotective effects of Met in a rat model of HFD/STZ-induced DN. Untreated DN rats developed marked metabolic and renal abnormalities, including hyperglycemia, insulin resistance, proteinuria, and reduced creatinine clearance. Our data also showed a substantial increase in serum cystatin C, a sensitive endogenous marker of GFR that has demonstrated diagnostic value in DN [49,50]. Additionally, the DN group exhibited significant increases in renal KIM-1 and NGAL together with urinary β2-microglobulin. KIM-1 and NGAL are sensitive biomarkers of tubular epithelial injury [5,6,51], whereas increased urinary β2-microglobulin reflects impaired proximal tubular reabsorption in the context of diabetes-induced renal damage [52,53]. Consistent with these functional and biochemical abnormalities, H&E-stained renal sections from the DN group revealed prominent tubular damage, interstitial edema, and inflammatory infiltration, aligning with well-recognized tubulointerstitial structural abnormalities associated with diabetic nephropathy [2,3,47,48]. Collectively, these findings demonstrate concurrent glomerular and tubular impairment and closely recapitulate the characteristic renal phenotype of DN, in which progressive urinary protein loss and declining filtration capacity accompany tubulointerstitial injury [1,2,3,5,6].

Untreated DN rats also showed markedly elevated serum AGEs together with increased renal MDA, protein carbonyl, 8-OHdG, and iNOS, accompanied by significant reductions in GSH, GPx1, CAT, and SOD. These findings are consistent with earlier reports demonstrating that oxidative and nitrosative imbalance is a central pathogenic component of DN and contributes to progressive renal cellular injury [4,8,9]. Renal Nrf2 concentrations and Hmox1 expression were also markedly reduced in the DN group, indicating impairment of endogenous antioxidant and cytoprotective responses [8,54]. Interestingly, Keap1 expression, which negatively regulates Nrf2 under basal conditions, was concurrently decreased. Rather than indicating a simple linear suppression of Nrf2 signaling, the combined reductions in Keap1, Nrf2, and Hmox1 suggest broader dysregulation of the Keap1/Nrf2/HO-1 redox-regulatory system during DN [8,10]. These alterations were further supported by the correlation analysis, in which oxidative-stress and renal-injury markers showed strong positive associations, whereas Nrf2 and endogenous antioxidant defenses were inversely related to indices of renal injury. Together, our data further support the notion that chronic uncontrolled hyperglycemia triggers and sustains renal injury through enhanced AGE formation and persistent oxidative stress [4,7,55].

Renal inflammation was also prominent in untreated DN rats, as demonstrated by marked elevations in TNF-α, IL-1β, IL-6, TLR4, and NF-κB1 together with reduced IL-10. These findings are consistent with the established role of TLR4/NF-κB-mediated inflammatory signaling in DN, whereby persistent metabolic and oxidative stress promotes cytokine production and sustains renal tissue injury [4,10,14]. The DN group also showed increased Mapk1 expression, further supporting disturbance of stress-responsive signaling mechanisms involved in inflammatory and fibrotic progression in diabetic kidney disease [11,56]. Concurrently, renal TGF-β1 was markedly elevated, consistent with its central profibrotic role in extracellular matrix accumulation and progressive renal fibrosis [2,19]. This inflammatory and profibrotic milieu was accompanied by increased BAX and Caspase-3 and reduced BCL-2, indicating enhanced renal apoptosis, which contributes to progressive structural deterioration in DN [5,6,24]. These abnormalities were strongly interconnected in the untreated DN group, where Pearson analysis showed positive associations among inflammatory, profibrotic, apoptotic, and renal-injury markers, whereas IL-10 and BCL-2 were inversely related to indices of renal damage. Consistently, Mantel analysis identified Mapk1 as the gene most strongly associated with injury-related biomarker clusters. Collectively, our findings support the notion that the pathogenesis and progression of DN involve intricate and interrelated pathogenic mechanisms, whereby chronic uncontrolled hyperglycemia promotes persistent oxidative stress and pro-inflammatory signaling that subsequently aggravate profibrotic responses, cellular apoptosis, and progressive renal damage [2,4,7,14,19,24,57].

Met monotherapy significantly improved glycemic control and renal function in DN rats, as demonstrated by reductions in fasting blood glucose, fasting insulin, HbA1c, HOMA-IR, and serum AGEs, together with amelioration of serum and urinary indices of renal dysfunction. These findings are consistent with the established antihyperglycemic and renoprotective actions of Met in experimental and clinical DN [12,15,16,17,18,23]. The observed improvements in renal biochemical profiles were accompanied by marked reductions in renal KIM-1, NGAL, and TGF-β1 concentrations and attenuation of renal histopathological abnormalities, further supporting protection against both functional and structural renal injury [15,16,19].

Met treatment also significantly reduced TNF-α, IL-1β, IL-6, TLR4, NF-κB1, iNOS, protein carbonyl, MDA, and 8-OHdG, while increasing IL-10, Nrf2, GSH, CAT, and SOD, in agreement with previous reports that Met attenuates inflammatory and oxidative-stress pathways implicated in the progression of DN [15,19,20,21,22]. Similarly, BAX and Caspase-3 declined, whereas BCL-2 increased, indicating attenuation of renal apoptosis [58,59]. At the transcriptional level, Met decreased Mapk1 and increased Akt1 and Hmox1, whereas Keap1 remained unchanged relative to untreated DN, suggesting partial modulation of the investigated stress-response and cytoprotective mechanisms [11,18]. Consistent with these findings, Pearson and Mantel analyses showed weaker associations between injury-related biomarker clusters and Mapk1, alongside relatively greater associations involving Akt1 and Hmox1 than in untreated DN animals. Nevertheless, the overall response to Met remained partial, since many metabolic, inflammatory, oxidative-stress, renal-injury, and apoptotic markers remained significantly abnormal compared with NC animals. These residual abnormalities support the need for complementary therapeutic approaches capable of enhancing the renoprotective effects of Met by more effectively targeting persistent oxidative stress and inflammatory signaling in DN [4,7,8,9,57].

Although fasting blood glucose, HbA1c, and fasting insulin were comparable between the EDA/Met co-treatment groups and Met monotherapy, addition of both low- and high-dose EDA significantly reduced HOMA-IR and serum AGEs. The available experimental literature regarding the anti-diabetic effects of EDA is limited and appears to be model-dependent. In a multiple-low-dose STZ model of type 1 diabetes, EDA attenuated hyperglycemia primarily by preserving pancreatic β-cell integrity and insulin production against oxidative injury rather than by directly regulating glucose metabolism [60]. By contrast, several studies in animals with established diabetes or diabetic complications have reported substantial tissue-protective effects of EDA monotherapy (10 mg/kg) without significant reductions in blood glucose levels, including experimental diabetic nephropathy and cardiomyopathy models [61,62,63]. Consistent with these observations, the present findings do not support a direct hypoglycemic effect of EDA in established DN [61,62,63], but suggest that its addition may enhance Met-mediated improvements in insulin resistance and glycation burden. Nevertheless, because the current study did not include a DN group treated with EDA alone, its independent effects on insulin sensitivity and glucose homeostasis cannot be determined. Further studies incorporating EDA monotherapy, glucose- and insulin-tolerance testing, pancreatic β-cell assessment, and direct analysis of insulin-signaling pathways in experimental type 2 diabetes are required to determine whether EDA exerts independent insulin-sensitizing or glucose-regulatory actions.

However, addition of EDA at both doses significantly enhanced the renoprotective effects of Met by further mitigating serum and urinary indices of renal dysfunction, reducing renal KIM-1, NGAL, and TGF-β1 concentrations, and showing greater preservation of renal histological architecture. The higher EDA dose generally produced the most consistent improvements, with several renal functional, injury, and histopathological parameters approaching values observed in NC animals. Our data are consistent with previous experimental evidence demonstrating that EDA attenuates diabetic renal dysfunction and structural injury despite limited effects on blood glucose [61], and with studies showing protective actions of EDA against oxidative and inflammatory tissue injury in other diabetic complications [62,63,64,65,66].

The enhanced renoprotection observed with EDA co-treatment was accompanied by marked attenuation of renal redox imbalance. Addition of both low- and high-dose EDA to Met further reduced renal iNOS and 8-OHdG and significantly increased Nrf2, GPx1, CAT, and SOD, whereas the higher EDA dose additionally lowered protein carbonyl concentrations relative to Met monotherapy. These findings are consistent with the potent free-radical-scavenging activity of EDA [25] and with previous studies showing that EDA attenuates oxidative tissue injury through enhancement of Nrf2-associated antioxidant responses, including in experimental diabetic cardiomyopathy [62,67,68]. At the transcriptional level, the higher EDA dose significantly increased *Hmox1* and *Keap1* expression relative to Met alone, whereas the lower dose did not significantly alter either gene. Since Keap1 is a negative regulator of Nrf2, restoration of *Keap1* expression toward control values should not itself be interpreted as evidence of Nrf2 activation. Rather, the concomitant increase in renal Nrf2, *Hmox1* expression, and endogenous antioxidant enzymes suggests broader restoration of redox-regulatory homeostasis with high-dose EDA/Met co-treatment [8,28,54,69]. Consistent with this interpretation, the correlation and Mantel analyses showed stronger associations of *Hmox1* and *Akt1* with protective antioxidant profiles in the EDA co-treatment groups, particularly with the higher dose, although these relationships remain associative and do not establish pathway activation. Future mechanistic studies should therefore evaluate Nrf2 nuclear translocation, HO-1 protein expression, and AKT phosphorylation, together with targeted loss- and gain-of-function approaches involving the Keap1/Nrf2/HO-1 axis and AKT signaling in renal cells under normal- and high-glucose conditions, to determine their direct contribution to EDA-mediated cytoprotection.

EDA co-treatment also produced substantial additional anti-inflammatory and antiapoptotic effects relative to Met monotherapy. Both EDA doses further reduced renal TLR4, NF-κB1, TNF-α, IL-1β, and IL-6 while increasing IL-10, consistent with previous evidence that EDA suppresses NF-κB-associated inflammatory responses in diabetic and other oxidative-injury models [32,64,65]. The higher EDA dose additionally reduced *Mapk1* expression relative to Met alone, supporting a potential involvement of MAPK-associated stress signaling in the enhanced anti-inflammatory response [35,66]. Concurrent reductions in TGF-β1 with both co-treatment regimens also suggest attenuation of profibrotic signaling, in agreement with reports that EDA mitigates TGF-β1-associated tissue fibrosis, including in experimental diabetes [34,63]. These anti-inflammatories and profibrotic improvements were accompanied by lower BAX and Caspase-3 and increased BCL-2, indicating enhanced protection against renal apoptosis [30,65,70]. Consistent with these findings, correlation analyses showed progressive weakening of the relationships among inflammatory, apoptotic, and renal-injury markers following EDA co-treatment, while Mantel analysis showed weaker *Mapk1*-centered associations, particularly with the higher dose. Collectively, these findings suggest that attenuation of TLR4/NF-κB1- and MAPK-associated inflammatory responses, together with reduced profibrotic and apoptotic activity, may contribute to the additional renoprotection afforded by EDA/Met co-treatment. However, direct pathway involvement remains to be established by assessing MAPK phosphorylation, NF-κB nuclear translocation, and pathway-specific loss- or gain-of-function interventions. Taken together, our findings and previous experimental evidence suggest that the additional renoprotective effects of EDA when combined with Met may be mediated predominantly through attenuation of oxidative and inflammatory injury rather than through further enhancement of glycemic regulation [25,61,62,63,64,65,66].

### Study Limitations

Several limitations of the present study should be acknowledged. First, the experimental design did not include diabetic groups treated with EDA alone. Therefore, the independent metabolic and renoprotective effects of EDA could not be determined, and the greater efficacy observed with EDA/Met co-treatment cannot establish pharmacological synergy. Second, only male rats were included; therefore, potential sex-specific differences in the response to EDA/Met co-treatment were not assessed, which may limit the generalizability of the findings to females [37,38]. Third, the relatively modest sample size and the use of a single HFD/STZ-induced rat model may limit the broader translational applicability of the findings to the heterogeneous clinical spectrum of human DN [41]. Fourth, the mechanistic interpretation was based partly on renal gene expression and tissue concentrations of signaling-related proteins, without direct assessment of pathway activation through phosphorylation status, nuclear translocation, or pathway-specific inhibition. Accordingly, the observed changes in *Mapk1*, *Akt1*, *Keap1*, and *Hmox1*, together with Nrf2, TLR4, and NF-κB1, should be interpreted as associative rather than as definitive evidence of pathway activation or suppression. Fifth, the Pearson correlation and Mantel analyses identify coordinated relationships among biomarkers and genes but cannot establish the direction or causality of these interactions. Finally, although EDA was administered in vivo and samples were collected after treatment, no dedicated in vitro spiking or recovery experiments were performed to formally exclude potential analytical interference of EDA with the biochemical or ELISA assays.

## 5. Conclusions

In conclusion, Met monotherapy provided partial metabolic and nephroprotective effects against established DN, whereas addition of EDA produced further improvements in renal function, tubular injury, histopathological damage, oxidative stress, inflammation, fibrosis, and apoptosis, with the higher EDA dose showing the most consistent benefit. The enhanced renoprotection was associated with improvement of Nrf2-associated antioxidant defenses, attenuation of TLR4/NF-κB1- and MAPK-associated inflammatory responses, reduced TGF-β1 and apoptotic activity, and modulation of *Mapk1*, *Akt1*, *Keap1*, and *Hmox1*. However, these molecular findings remain associative and do not establish direct pathway activation or pharmacological synergy.

Future studies should therefore include multiple-dose EDA monotherapy protocols to precisely define its independent metabolic and renoprotective actions and to distinguish additive from synergistic effects. Additional experiments should also evaluate glucose and insulin tolerance, pancreatic β-cell function, AKT and MAPK phosphorylation, Nrf2 nuclear translocation, HO-1 protein expression, NF-κB nuclear localization, and pathway-specific loss- or gain-of-function approaches, both in vitro and in vivo. Validation in both sexes, larger cohorts, and complementary experimental models is also warranted before considering clinical translation.

## Figures and Tables

**Table 1:** Glycemic, insulin-resistance, and glycation profiles across experimental groups.

Parameter	Groups						One-Way ANOVA *p*-Value (F-Value)
	NC	EDA10	DN	DN + Met	DN + ME5	DN + ME10	
**Fasting Glucose** **(mg/dL)**	98.50 ± 6.16	102.50 ± 5.18	309.38 ± 17.35 ***	153.12 ± 13.79 **^,###^	140.62 ± 9.21 **	138.25 ± 9.59 **	<0.0001 (396.19)
**HbA1c (%)**	5.05 ± 0.19	5.17 ± 0.26	12.55 ± 0.47 ***	7.15 ± 0.37 **^,###^	6.64 ± 0.41 **	6.77 ± 0.32 **	<0.0001 (497.40)
**Fasting Insulin** **(μIU/mL)**	2.06 ± 0.26	2.19 ± 0.30	10.82 ± 2.08 ***	4.40 ± 0.31 **^,###^	4.11 ± 0.54 **	4.30 ± 0.62 **	<0.0001 (94.10)
**HOMA-IR**	0.50 ± 0.06	0.54 ± 0.07	8.25 ± 1.31 ***	1.66 ± 0.43 **^,###^	1.43 ± 0.46 *^,††^	1.48 ± 0.41 ^‡‡^	<0.0001 (182.78)
**AGEs (ng/mL)**	164.25 ± 16.48	166.88 ± 11.32	790.62 ± 38.03 ***	290.60 ± 25.63 **^,###^	213.00 ± 18.45 *^,††^	182.88 ± 14.51 ^‡‡‡^	<0.0001 (937.53)

Values are presented as mean ± SD (*n* = 8 rats/group). Differences among groups were analyzed using one-way ANOVA followed by Tukey’s multiple-comparison post hoc test. The final column reports the overall one-way ANOVA *p* value, with the corresponding F value in parentheses. Superscript symbols indicate Tukey-adjusted pairwise comparisons: * denotes comparison with NC; ^#^ denotes DN versus DN + Met; ^†^ denotes DN + Met versus DN + ME5; and ^‡^ denotes DN + Met versus DN + ME10. One, two, and three identical symbols indicate *p* < 0.05, *p* ≤ 0.01, and *p* ≤ 0.001, respectively. Absence of a comparison-specific symbol indicates *p* > 0.05. NC, negative control; EDA10, non-diabetic control treated with edaravone (10 mg/kg); DN, untreated diabetic nephropathy; DN + Met, diabetic nephropathy treated with metformin (300 mg/kg); DN + ME5, diabetic nephropathy treated with metformin plus edaravone (5 mg/kg); DN + ME10, diabetic nephropathy treated with metformin plus edaravone (10 mg/kg).

**Table 2:** Renal inflammatory, oxidative-stress, and antioxidant markers across experimental groups.

Parameter	Groups						One-Way ANOVA *p*-Value (F-Value)
	NC	EDA10	DN	DN + Met	DN + ME5	DN + ME10	
**TNF-α (pg/mL)**	40.62 ± 8.07	36.62 ± 6.70	195.12 ± 8.58 ***	98.00 ± 8.73 **^,###^	73.25 ± 7.55 **^,†††^	42.25 ± 10.90 ^‡‡‡,$$$^	<0.0001 (407.09)
**IL-1β (pg/mL)**	45.25 ± 6.18	39.12 ± 5.67	187.25 ± 22.91 ***	95.12 ± 9.96 **^,###^	63.50 ± 6.50 *^,†††^	42.10 ± 7.39 ^‡‡‡,$$^	<0.0001 (197.96)
**IL-6 (pg/mL)**	57.50 ± 6.59	54.25 ± 7.57	181.88 ± 15.76 ***	97.00 ± 9.41 **^,###^	74.50 ± 6.87 *^,†††^	60.50 ± 7.11 ^‡‡‡^	<0.0001 (213.35)
**TLR4 (ng/mL)**	1.94 ± 0.58	1.85 ± 0.41	6.92 ± 1.08 ***	3.46 ± 0.85 **^,###^	2.62 ± 0.42 *^,†††^	2.04 ± 0.35 ^‡‡‡^	<0.001 (69.71)
**NF-κB1 (ng/mL)**	2.55 ± 0.80	2.36 ± 0.62	8.65 ± 1.46 ***	5.10 ± 0.99 **^,###^	3.25 ± 0.49 *^,†††^	2.45 ± 0.61 ^‡‡‡^	<0.001 (61.30)
**IL-10 (pg/mL)**	37.62 ± 7.17	38.88 ± 6.38	18.62 ± 2.77 ***	34.25 ± 3.06 **^,###^	39.38 ± 4.24 ^††^	40.62 ± 4.96 ^‡‡^	<0.0001 (21.60)
**Nrf2 (ng/mL)**	18.80 ± 4.55	17.82 ± 4.26	8.84 ± 2.10 ***	11.79 ± 2.36 *^,###^	16.06 ± 2.36 ^††^	18.13 ± 2.03 ^‡‡^	<0.001 (12.93)
**GSH (mg/g)**	31.88 ± 6.01	32.88 ± 5.51	16.88 ± 4.16 ***	27.25 ± 4.62 ^###^	28.88 ± 3.91	30.62 ± 2.67	<0.0001 (12.85)
**GPx1 (µg/mg)**	6.79 ± 1.41	7.26 ± 1.18	4.11 ± 0.90 ***	5.21 ± 0.82 *	5.81 ± 0.52 ^†^	6.28 ± 1.44 ^‡^	<0.0001 (8.60)
**CAT (U/mg)**	242.62 ± 21.10	247.62 ± 25.58	188.50 ± 18.45 ***	211.50 ± 24.59 *^,###^	244.12 ± 23.35 ^††^	253.88 ± 11.01 ^‡‡^	<0.0001 (11.66)
**SOD (U/mg)**	51.25 ± 9.65	50.50 ± 7.89	27.88 ± 5.59 ***	46.25 ± 3.69 *^,###^	51.62 ± 5.73 ^††^	54.75 ± 5.04 ^‡‡^	<0.0001 (17.76)
**iNOS (ng/mg)**	0.88 ± 0.27	0.85 ± 0.24	3.85 ± 0.46 ***	2.90 ± 0.16 **^,###^	2.27 ± 0.27 **^,††^	1.34 ± 0.27 *^‡‡‡,$$$^	<0.0001 (136.78)
**Carbonyl-protein (ng/mg)**	0.77 ± 0.31	0.77 ± 0.41	5.24 ± 0.88 ***	2.31 ± 0.41 **^,###^	1.96 ± 0.49 **	0.71 ± 0.23 ^‡‡‡,$$$^	<0.0001 (97.82)
**MDA (nmol/g)**	23.38 ± 4.50	23.75 ± 3.99	46.38 ± 7.01 ***	30.88 ± 4.73 *^,###^	28.10 ± 3.85	27.38 ± 3.34	<0.0001 (26.04)
**8-OHdG (ng/mg)**	0.88 ± 0.15	0.92 ± 0.32	2.89 ± 0.22 ***	2.31 ± 0.34 **^,##^	1.80 ± 0.41 **^,†^	1.16 ± 0.32 ^‡‡,$$^	<0.0001 (56.86)

Values are presented as mean ± SD (*n* = 8 rats/group). Differences among groups were analyzed using one-way ANOVA followed by Tukey’s multiple-comparison post hoc test. The final column reports the overall one-way ANOVA *p* value, with the corresponding F value in parentheses. Superscript symbols indicate Tukey-adjusted pairwise comparisons: * denotes comparison with NC; ^#^ denotes DN versus DN + Met; ^†^ denotes DN + Met versus DN + ME5; ^‡^ denotes DN + Met versus DN + ME10; and ^$^ denotes DN + ME5 versus DN + ME10. One, two, and three identical symbols indicate *p* < 0.05, *p* ≤ 0.01, and *p* ≤ 0.001, respectively. Absence of a comparison-specific symbol indicates *p* > 0.05. NC, negative control; EDA10, non-diabetic control treated with edaravone (10 mg/kg); DN, untreated diabetic nephropathy; DN + Met, diabetic nephropathy treated with metformin (300 mg/kg); DN + ME5, diabetic nephropathy treated with metformin plus edaravone (5 mg/kg); DN + ME10, diabetic nephropathy treated with metformin plus edaravone (10 mg/kg).

## Data Availability

The data that support the findings of this study are available within the article. Furthermore, inquiries can be directed to the corresponding author upon reasonable request. The authors affirm that all data were generated internally, that no external paper production services were utilized.

## Supplementary Materials

The following supporting information can be downloaded at: https://www.mdpi.com/article/10.3390/biology15211988/s1. Figure S1: Overall experimental design and animal allocation; Table S1: Primer sequences used for quantitative real-time PCR.
