# Supplementary material for: Edaravone Improves the Efficacy of Metformin Against Diabetic Nephropathy by Attenuating Inflammation, Oxidative Stress, and Apoptosis with Associated Modulation of MAPK/NF-κB1 and AKT/NRF2 Pathways in Rats
Source: Biology (Basel). 2026 Nov 3;15(21):1988. doi: 10.3390/biology15211988 (PMC13761653; doi:10.3390/biology15211988)

Supplementary Figure S1. Experimental Study Design and Animal Flow

Induction of type 2 diabetes mellitus (T2DM) and diabetic nephropathy (DN), group allocation, and therapeutic intervention

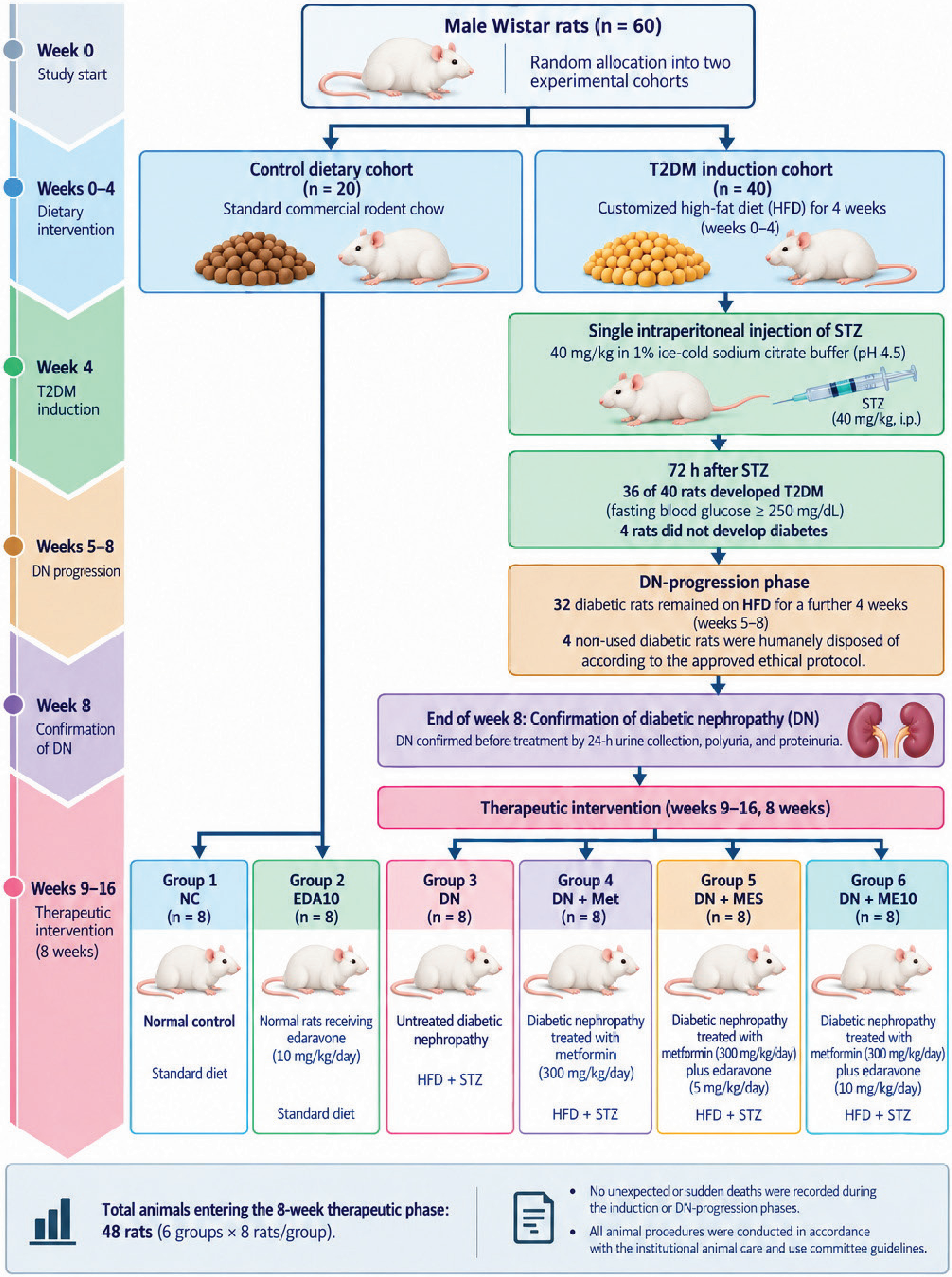

Supplement: Supplementary file 1 [file biology-15-01988-s001.zip › Figure S1.pdf]
