# Supplementary material for: Edaravone Improves the Efficacy of Metformin Against Diabetic Nephropathy by Attenuating Inflammation, Oxidative Stress, and Apoptosis with Associated Modulation of MAPK/NF-κB1 and AKT/NRF2 Pathways in Rats
Source: Biology (Basel). 2026 Nov 3;15(21):1988. doi: 10.3390/biology15211988 (PMC13761653; doi:10.3390/biology15211988)
Supplement: Supplementary file 1 [file biology-15-01988-s001.zip › Table S1.pdf]

Supplementary Table S1. Primer sequences used for the qRT-PCR detection of rat *Mapk-1, Akt1, Keap-1,* and *HO-1* mRNAs in renal samples, including the corresponding GenBank accession numbers, annealing temperatures, and amplicon sizes. *Actb* (*β-actin*) was used as the internal reference gene.

Genes	GenBank accession number	Oligonucleotide sequence (5′–3′)	Annealing temperature (C^0)	**Amplicon size (bp)**
MAPK-1	NM_053842.2	F - CTC GTA CAT CGG AGA AGG CG<br>R - CAT GTC TGA AGC GCA GTA GGA	60	158
AKT1	NM_033230.3	F- GCC ACG GAT ACC ATG AAC GA<br>R- AGT TGT TGA GTG GGG ACT CG	60	175
Keap-1	NM_057152.2	F- TAC AAC CCC ATG ACC AAC CAG<br>R- CCA CTC GTC TCG ATC TGG C	60	165
HO-1	NM_012580.2	F- CCA GAG TTT CCG CCT CCA AC<br>R- TCA AGG CCT CGG ACA AAT CC	60	163
Actb (β-Actin)	NM_031144.3	F- CCC GCG AGT ACA ACC TTC TT<br>R- CGC AGC GAT ATC GTC ATC CA	60	83
GAPDH	NM_017008.4	F- GCA TCT TCT TGT GCA GTG CC<br>R- GAG AAG GCA GCC CTG GTA AC	60	105

Mapk1, mitogen-activated protein kinase 1; *Akt1*, AKT serine/threonine kinase 1; *Keap-1*, Kelch-like ECH-associated protein 1; *HO-1*, heme oxygenase 1 (HO-1); *Actb*, actin beta (β-actin; internal reference gene).
